# When the gatekeeper falls: developmental vulnerability of the thalamic reticular nucleus in neonatal and pediatric hypoxic-ischemic brain injury

**DOI:** 10.3389/fnsys.2026.1753562

**Published:** 2026-03-10

**Authors:** Kuangfu Hsiao

**Affiliations:** 1 Children's National Hospital, Washington, DC, United States; 2 George Washington University School of Medicine and Health Sciences, Washington, DC, United States

**Keywords:** cardiac arrest, chloride homeostasis, connexin-36, developmental neurophysiology, GABAA receptor, hypoxia–ischemia, pediatrics, thalamic reticular nucleus

## Abstract

The thalamic reticular nucleus orchestrates thalamocortical oscillations and sensory gating. Its early development features a unique confluence of depolarizing GABA signaling, immature chloride regulation, and transient electrical coupling via connexin-36 gap junctions. These developmental specializations, essential for synchronizing cortical maturation, also render thalamocortical networks vulnerable to hypoxic–ischemic insults such as perinatal asphyxia or pediatric cardiac arrest. Following cellular ATP depletion, rapid chloride imbalance eliminates fast synaptic inhibition, permitting abnormal network activity to propagate via gap-junction coupling that persists when chemical inhibition collapses. The resulting electrical hypersynchrony, exacerbated by depolarizing GABAergic currents and impaired chloride extrusion, promotes excitotoxicity and thalamocortical dysrhythmia. This review synthesizes recent evidence to establish a framework that accounts for the selective vulnerability of the immature brain. Understanding these mechanisms may inform strategies to preserve developmental integrity and promote circuit resilience after pediatric asphyxial events.

## 1 Introduction

### 1.1 Clinical burden of neonatal hypoxic–ischemic injury and the thalamus as a vulnerable structure

Hypoxia-ischemia in infants and pediatric patients causes oxygen and blood flow deprivation to the developing brain (Berger and Garnier, 2000). This condition often stems from birth asphyxia or postnatal cardiac arrest (Douglas-Escobar and Weiss, 2015; Iordanova et al., 2017). It leads to neonatal encephalopathy and lifelong disabilities like sensory, motor, and cognitive deficits (Abusaleem et al., 2024; Huntingford et al., 2024; Kurinczuk et al., 2010; Schreglmann et al., 2020; Van Handel et al., 2007). Therapeutic hypothermia has reduced mortality (Azzopardi et al., 2009; Gonzalez and Ferriero, 2009; Gunn et al., 1998; Shankaran et al., 2005; Yenari and Han, 2012). However, many survivors exhibit persistent impairments from delayed damage cascades, such as seizures (El-Dib et al., 2023; Franck et al., 2020; Gluckman et al., 2005). These delayed processes unfold after the immediate metabolic insult. These secondary and tertiary cascades implicate disrupted inhibitory circuitry and network dynamics (Azzopardi et al., 1989; Davidson et al., 2012a; Davidson et al., 2021; Siesjö, 1984; Thornton et al., 2018). Indeed, neuroimaging and pathological studies consistently find that subcortical structures, including the basal ganglia, thalamus, and hippocampus, corpus callosum are key sites of injury hours to weeks following initial hypoxic–ischemic insult in neonates (Lemmon et al., 2017; Miller et al., 2005; Spencer et al., 2023; Volpe, 2009). Diffusion-weighted MRI of neonates with perinatal asphyxia demonstrates early signal abnormalities within the ventrolateral and posterior thalamic nuclei in roughly 70% of affected infants, correlating with the severity of encephalopathy and subsequent neurodevelopmental impairment (Barnett et al., 2002; Bonkowsky and Son, 2018; Perez et al., 2013). Pediatric survivors show persistent sensory and cognitive impairments in 40–60% of cases, reflecting long-term thalamocortical dysfunction as a major neurological sequela of hypoxic–ischemic brain injury (Donoghue et al., 2005; El-Seify et al., 2023; Mir et al., 2022; Nolan et al., 2008; Scheibe et al., 2020; Snider et al., 2022). These clinical findings highlight the thalamus’ role in perinatal hypoxia–ischemic damage and implicate disrupted thalamocortical signaling as a major determinant of persistent neurological disability.

### 1.2 The thalamic reticular nucleus as thalamocortical circuits’ gatekeeper

The thalamic reticular nucleus (TRN) occupies an anatomical position that underpins its crucial role in circuit maturation while its functions are predisposed to selective vulnerability under hypoxic–ischemic conditions (Böttiger et al., 1998; Geocadin et al., 2000; Kawai et al., 1995; Nolan et al., 2008; Radovsky et al., 1997; Ross and Graham, 1993; Shoykhet et al., 2012). The TRN is a thin shell of GABAergic neurons wraps around the lateral aspect of the dorsal thalamus, positioned at the intersection of ascending thalamocortical and descending corticothalamic pathways where it receives excitatory collaterals from both projections and returns GABAergic inhibition to thalamic relay neurons (Harris, 1987; Houser et al., 1980; Jones, 1975; Kim et al., 1997; Zikopoulos and Barbas, 2007). Naturally, it plays a key role in shaping patterns of thalamocortical oscillation and controls the flow of sensory information to the cortex (Bazhenov et al., 1999; Cox et al., 1997; Crabtree et al., 1998; Crick, 1984; Macdonald et al., 1998). Hypoxic–ischemic stress disrupts these oscillations (Failor et al., 2010; Shoykhet et al., 2012; Wikström et al., 2012). This review draws attention to the studies of region-specific developmental pattern of TRN, with the aim of linking it to the nucleus’s greater susceptibility to ischemic insults compared to other developing brain regions.

### 1.3 Scope and organization of this review

This review begins with the TRN pathology during neonatal brain injury from low oxygen (hypoxic-ischemia) and child cardiac arrest from lack of air (asphyxia) in clinical settings and in experimental models (*Section 2*). We summarize the cellular and functional maturation of normative TRN during perinatal and postnatal periods, with a focus on cation-chloride cotransporters and electrical synapses (*Section 3*). We then examine the mechanisms that disrupt multiple intra-TRN signaling pathways during hypoxic–ischemic injury from events such as pediatric asphyxial cardiac arrest, linking these pathway disruptions to underlying chloride dysregulation (*Section 4 and 5*). Finally, we discuss clinical implications and future therapeutic directions (*Section 6*).

## 2 Thalamic reticular nucleus pathology in clinical and experimental models of neonatal injury

### 2.1 Human neuropathology following perinatal asphyxia and cardiac arrest

Neuroimaging studies using MRI have profoundly advanced the understanding of hypoxic–ischemic injury in neonates and children, consistently revealing that the thalamus and its connected subcortical structures are principal targets of injury (Alderliesten et al., 2013; Hayashi et al., 1991; Leech and Alvord, 1977; Schneider et al., 1975). Early diffusion-weighted and volumetric MRI analyses in term infants with hypoxic–ischemic encephalopathy demonstrate two characteristic injury patterns: a basal ganglia–thalamus (BGT) pattern involving the ventrolateral thalamus, posterior putamen, and perirolandic cortex, and a watershed pattern predominantly affecting parasagittal white matter and overlying cortex (De Vries and Groenendaal, 2010; Miller et al., 2005). The BGT pattern is typically observed following acute, profound asphyxia and is associated with severe motor impairment and dyskinetic cerebral palsy, whereas the watershed pattern follows partial, prolonged hypoxia and more often leads to cognitive and behavioral deficits detected later in childhood. Quantitative MRI approaches have extended these findings: Kebaya et al. showed that, despite therapeutic hypothermia, neonates with hypoxic–ischemic encephalopathy scanned within the first week of life exhibit significantly smaller bilateral thalamic, basal ganglia, hippocampal, and cerebellar volumes compared to healthy controls, with volumetric loss correlating with the severity of encephalopathy (Kebaya et al., 2023). In older children, Tierradentro-García et al. provided further anatomical resolution, demonstrating that 66% of cases show involvement of the thalamic relay nuclei supplied by the thalamogeniculate arteries, most commonly in those with the BGT pattern, while posterior pulvinar injury predominates in the watershed pattern (Tierradentro-Garcia et al., 2023). However, given the TRN’s critical role in modulating thalamic relay output, the functional consequences of general thalamic injury likely include disrupted reticular inhibitory control (Chakkarapani et al., 2025; Govaert et al., 2025)underscores the need to examine the microscopic organization of thalamic inhibitory systems.

Neuropathological examinations following perinatal asphyxia and pediatric cardiac arrest cases revealed an age-related pattern in TRN susceptibility. In the seminal report by (Ross and Graham, 1993), selective neuronal loss within the TRN was identified in 3 of 6 pediatric brains and 11 of 22 adult brains, demonstrating that the phenomenon spans the developmental spectrum. Notably, three pediatric cases (ages 1, 2.5, and 13 years) exhibited the most severe and extensive TRN injury, indicating that marked vulnerability can manifest early in life. Among adults, lesion severity was more heterogeneous, with several young and older adults showing widespread cortical and mediodorsal/ventrolateral thalamic necrosis accompanied by relative TRN sparing. Importantly, selective TRN loss persisted under conditions of intraoperative hypothermia (22–26 °C), leading authors to conclude that “moderate to profound intraoperative hypothermia is not sufficient to prevent the selective loss of TRN neurons following human cardiac arrest.” While autopsy studies demonstrate selective TRN injury in patients who survived for hours to weeks after cardiac arrest (Kinney et al., 1994; Ross and Graham, 1993), these observations cannot disambiguate whether the damage reflects immediate post-arrest neuronal death or delayed, subacute TRN degeneration occurring during recovery. Experimental validation in model systems therefore remains necessary, as discussed in the next section.

### 2.2 Animal models recapitulating selective reticular nucleus vulnerability

Experimental models in immature rodents have provided detailed characterization of the temporal evolution and mechanisms of reticular nucleus injury following hypoxia-ischemia (Charriaut-Marlangue et al., 2013; Titomanlio et al., 2015). A common neonatal hypoxic–ischemic model, carotid ligation plus hypoxia at postnatal day 7 (Rice-Vannucci model)(Rice et al., 1981), thalamic injury is well documented in the ventrobasal (i.e., somatosensory) thalamus and is delayed relative to forebrain injury, with apoptotic morphology emerging ~24 h after the insult (Martin et al., 1997; Northington et al., 2001). Molecular studies in this model show a Fas–caspase apoptotic cascade in ventrobasal thalamus that precedes caspase-3 activation and nuclear condensation, consistent with programmed cell death rather than early necrosis. Although the Rice–Vannucci preparation reproduces several features of human neonatal hypoxic–ischemic encephalopathy, injury of the TRN is not a consistent finding in this model (Towfighi et al., 1991). This contrasts with adult rat global ischemia models, in which the TRN, along with the hippocampal CA1, is one of the regions most vulnerable to ischemic insult (Blomqvist and Wieloch, 1985; Geocadin et al., 2000; Kawai et al., 1992; Ross and Duhaime, 1989; Smith et al., 1984).

**Figure 1:**
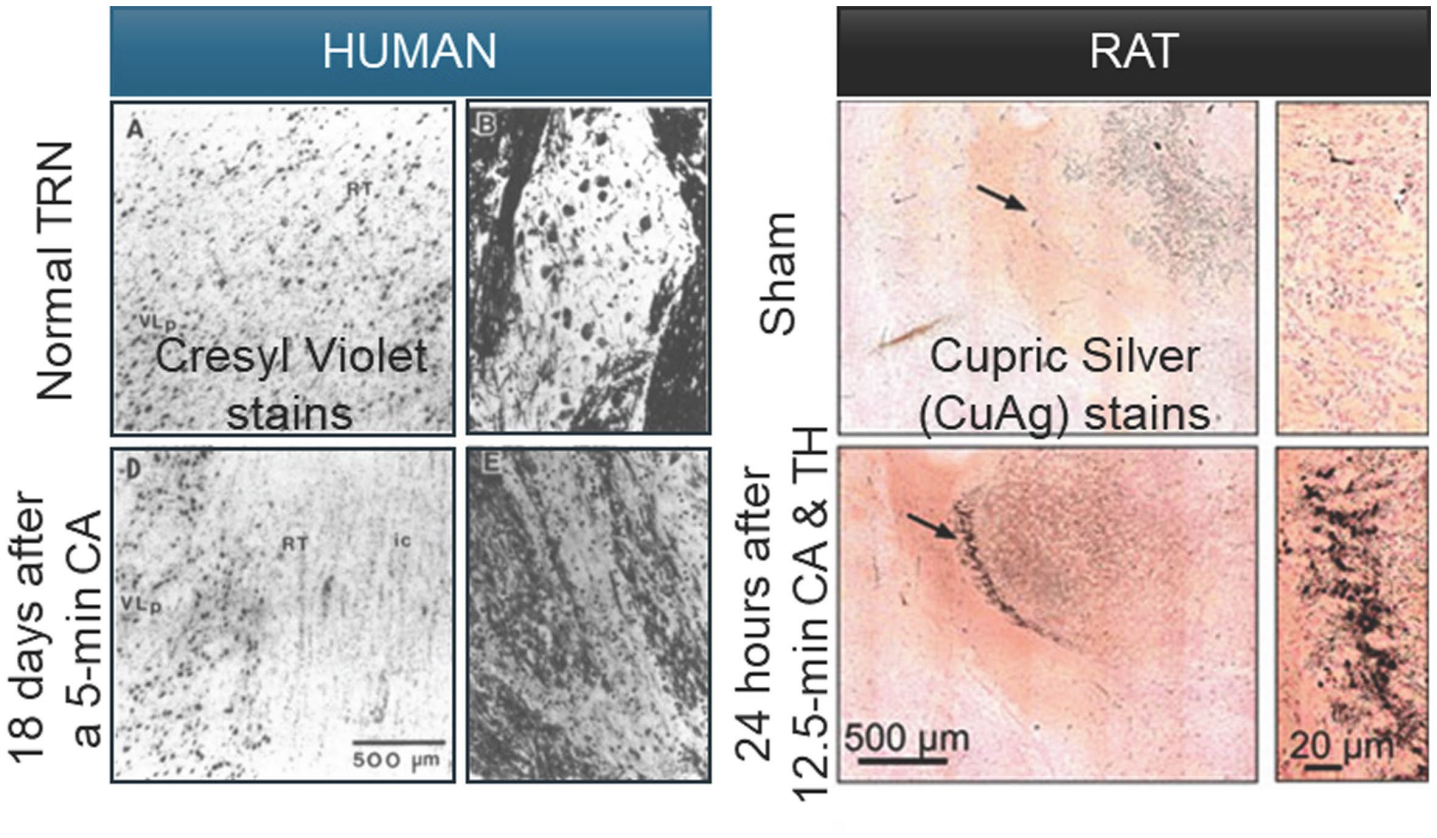
Selective neuronal degeneration in the thalamic reticular nucleus following asphyxial cardiac arrest in human and rat. Cupric-silver (CuAg) staining reveals degenerating neurons in the thalamic reticular nucleus 24 h after resuscitation from pediatric asphyxial cardiac arrest. Left panels: Human postmortem tissue from a cardiac arrest case demonstrates loss of one-third to one-half of the TRN neurons in affected regions. Right panels: Rat TRN sections from postnatal day 17–19 animals subjected to 12.5 min of asphyxial cardiac arrest show pronounced CuAg-positive neuronal profiles concentrated in intermediate and posterior TRN, replicating the selective regional vulnerability observed in human tissue. Sham-operated control animals exhibit minimal background staining and intact neuronal morphology throughout all TRN segments. The spatial concordance between human and experimental pathology validates the developmental asphyxial cardiac arrest model as a clinically faithful representation of selective TRN injury and establishes the ventral sensory sectors as key targets of post-resuscitation degeneration. Scale bars: 500 microns (100 microns zoom-in). CuAg, cupric-silver; TRN, thalamic reticular nucleus; CA, cardiac arrest, TH, therapeutic hypothermia [Human images adapted from Ross and Graham, 1993; rat images adapted from Ton et al., 2021, with authors’ permission].

Prolonged asphyxial cardiac arrest in developing rats provides anatomically and clinically faithful model of pediatric hypoxic–ischemic encephalopathy (Dietz et al., 2022; Fink et al., 2004). In recent study by Ton et al., postnatal day 17–19 rats underwent 11–12.5 min of cardiac arrest followed by full cardiopulmonary resuscitation and intensive post-arrest care (Figure 1, right panels) (Ton et al., 2021). This experimental pattern closely replicates the selective TRN degeneration observed in human postmortem tissue from pediatric cardiac arrest cases (Figure 1, left panels) (Ross and Graham, 1993). At twenty-four hours after resuscitation, histological analyses revealed pronounced and segment-specific degeneration within the TRN, accompanied by dense microglial activation. The injury showed a clear spatial gradient, with selective loss of GAD67-positive inhibitory neurons in the intermediate and posterior TRN, while the anterior segment remained structurally intact. These regions correspond, respectively, to somatosensory and auditory reticular sectors, whose degeneration was paralleled by synaptic loss in their thalamic targets, the ventroposteromedial (VPM) and medial geniculate (MGN) nuclei (Conley et al., 1991; Pierret et al., 2000; Pinault, 2004). In contrast, the mediodorsal nucleus, innervated by the anterior TRN, showed no degeneration. Mild post-arrest hypothermia (34 °C for 8 h) failed to attenuate either neuronal death or microglial accumulation, indicating that TRN injury in this context is hypothermia-resistant, consistent with earlier findings in both animal and human studies that TRN neurons remain refractory to temperature-based neuroprotection (Kawai et al., 1997; Ross and Graham, 1993).

Earlier work by using the same developmental asphyxial cardiac arrest model but shorter arrest durations (8–9 min) demonstrated persistent thalamic hyperexcitability without overt neuronal loss (Shoykhet et al., 2012). Recordings from VPM showed elevated spontaneous and evoked firing rates and prolonged receptive fields weeks after resuscitation, accompanied by microglial activation within the TRN. Middleton et al. extended these findings to the cortical level, showing that surviving animals exhibit long-term reductions in sensory-evoked responses and broader receptive fields in layer IV barrel cortex, despite histological preservation of both thalamic relay and reticular neurons (Middleton et al., 2017); while these measurements directly assess relay neuron function rather than TRN activity, the pattern of disinhibited relay firing combined with TRN microglial activation suggests impaired reticular inhibitory control. This series of experiments are particularly informative because they employed a unified experimental framework (i.e., pediatric asphyxial cardiac arrest model) under tightly controlled conditions, allowing direct comparison across injury severities and time points (Middleton et al., 2017; Shoykhet et al., 2012; Ton et al., 2021). Together, these studies establish a functional continuum across injury severities: brief, sublethal arrests cause enduring thalamocortical dysregulation arising from altered inhibitory control, whereas more prolonged or clinically representative arrests produce irreversible TRN degeneration. Importantly, ischemic stress results in thalamic hyperactivity and cortical hyporesponsiveness that manifest across different hierarchical network levels and evolve over distinct post-injury time courses, supporting the interpretation that progressive TRN dysfunction disrupts both thalamic gain and cortical precision as injury severity increases.

### 2.3 Electrophysiological signatures of reticular dysfunction after injury

Clinical electroencephalographic studies in neonates with hypoxic–ischemic encephalopathy consistently identify abnormal background patterns including burst-suppression (alternating periods of high-amplitude activity and electrical silence), low-voltage activity, and flat traces, within the first 24 to 36 h as strong predictors of adverse neurodevelopmental outcomes (Chakkarapani et al., 2025; Edoigiawerie et al., 2024). Quantitative analyses confirm that prolonged interburst intervals and attenuated amplitudes correlate with death or disability at 18–24 months, even in infants treated with therapeutic hypothermia (Dereymaeker et al., 2019). Despite cooling, severely abnormal EEG backgrounds or persistently high burst power in central and temporal regions remain associated with poorer cognitive and motor outcomes (Koskela et al., 2021), indicating enduring thalamocortical dysfunction.

Failure to develop sleep–wake cycling during the first postnatal days further marks disrupted thalamocortical regulation. The absence of cyclic transitions within 120 h of life shows high sensitivity for poor outcomes (Takenouchi et al., 2011). These EEG patterns reflect impaired maturation of oscillatory activity normally governed by reciprocal thalamic–reticular interactions (Fogerson and Huguenard, 2016; McCormick et al., 2015), consistent with evidence that sleep spindles and slow-wave activity depend on intact TRN–relay connectivity.

Experimental recordings in rodent models of asphyxial cardiac arrest confirm that thalamocortical networks exhibit persistent dysregulation following resuscitation. Thalamic relay neurons show elevated spontaneous and evoked firing rates, prolonged receptive fields, and increased inter-neuronal correlation weeks to months after injury, accompanied by microglial activation within the TRN (Middleton et al., 2017; Shoykhet et al., 2012). At the cortical level, surviving animals demonstrate reduced sensory-evoked responses and broader receptive fields in layer IV barrel cortex despite histological preservation of thalamic structures, indicating enduring functional impairment of thalamocortical signal processing (Middleton et al., 2017). These findings establish that subclinical to moderate hypoxic–ischemic insults produce persistent alterations in network dynamics characterized by loss of inhibitory precision and emergence of pathological synchrony.

## 3 Developmental organization: critical periods in thalamic reticular nucleus maturation

The formation of TRN circuitry follows a sequence of temporally ordered events spanning late embryonic to postnatal stages (Colonnese and Phillips, 2018; Grant et al., 2012; Pinault, 2004). During the perinatal period, thalamocortical axons reach the cortical plate and extend collaterals that innervate TRN neurons, establishing the first functional synapses (López-Bendito and Molnár, 2003; Mitrofanis and Baker, 1993; Moreno-Juan et al., 2017). Early postnatal activity is dominated by feedforward thalamocortical input, with corticothalamic feedback developing more gradually across the second postnatal week (Crair and Malenka, 1995; Evrard and Ropert, 2009; Lee et al., 2010; Warren and Jones, 1997). This temporal offset produces a transitional state in which TRN neurons receive strong ascending excitation but limited cortical modulation, a configuration that favors spontaneous network oscillations characteristic of the immature thalamocortex. The following section outlines current understanding of TRN synaptic organization as the anatomical substrate supporting these emergent thalamocortical dynamics.

### 3.1 Development of intra-TRN connectivity

A prominent feature of the developing TRN is the gradual strengthening of electrical coupling that supports coherent low-frequency population rhythms (Condorelli et al., 2000; Landisman et al., 2002; Liu and Jones, 2003), in contrast to the developmentally uncoupling of electrical synapses in cortical and hippocampal pyramidal neurons from glial cells (Hestrin and Galarreta, 2005). Intra-TRN electrical synapses, mediated by connexin 36 channels (Cx36), are prominent around birth and strengthen into the second postnatal week, supporting coherent low-frequency rhythms even when fast chemical synaptic transmission is pharmacologically silenced (Deans et al., 2001; Hormuzdi et al., 2001; Parker et al., 2009). This period of maximal electrical coupling, spanning approximately postnatal days 1–14 in rodents, creates functionally coupled ensembles of reticular neurons that can synchronize their activity independent of chemical neurotransmission (Lee et al., 2010; Warren and Jones, 1997; Zolnik and Connors, 2016). Functionally, this early electrically dominated state supports spontaneous thalamic oscillations preceding the establishment of adult-like spindle and slow-wave rhythms.

Overlapping with this period of electrical synapse development, GABAergic synapses within TRN follow a distinct developmental trajectory. Contrary to gradual strengthening, evidence indicates that intra-TRN GABAergic synapses are transiently abundant during early postnatal life and decline thereafter (Bentivoglio et al., 1991; Deleuze and Huguenard, 2006; Hou et al., 2016; Lam and Sherman, 2011). Optogenetic and transgenic studies further show that PV-positive TRN neurons form intra-TRN inhibitory synapses only until about postnatal day 14; beyond this age, deletion of vesicular GABA transporter in PV-positive neurons fails to reduce miniature IPSCs, implying that later inhibitory input arises from PV-negative TRN neurons or extrathalamic sources (Hou et al., 2016). The convergence of these findings suggests that synaptic coupling among TRN neurons decreases as electrical coupling strengthens, resulting in a developmental reorganization where early network synchrony relies on excitatory GABA and weak electrical coupling, while later coordination depends on extensive gap-junctional connectivity and inhibitory TRN-relay interactions.

### 3.2 Maturation of chloride homeostasis and the GABA polarity shift

The polarity of GABAergic signaling in reticular nucleus neurons undergoes developmental regulation through changes in chloride transporter expression (Bazhenov et al., 1999). In early postnatal life, high expression of the sodium-potassium-chloride cotransporter NKCC1 coupled with low expression of the potassium-chloride cotransporter KCC2 maintains elevated intracellular chloride concentrations such that GABA receptor activation produces chloride efflux and membrane depolarization (Achilles et al., 2007; Ben-Ari et al., 1989; Kaila et al., 2014; Rivera et al., 1999; Stein et al., 2004). This depolarizing GABA response is not simply a transitional state awaiting maturation but serves important developmental functions, contributing to calcium entry that guides synapse stabilization and activity-dependent refinement of connectivity.

Developing TRN neurons integrate several convergent vulnerability features: high Cx36-dependent electrical coupling, delayed KCC2 maturation that sustains depolarizing GABA action, and strong excitatory drive from early-maturing first-order thalamic nuclei (see *Section 3.4*). The combination predisposes these neurons at the intersection of chloride accumulation, electrically mediated propagation of depolarization, and enhanced glutamatergic load during hypoxia-ischemia. As a result, calbindin-rich ventral TRN sectors form a developmental and circuit-level hotspot for selective injury in neonatal and pediatric ischemic encephalopathy (Barthó et al., 2004; Klein et al., 2018; Schmidt et al., 2018). This prolonged immaturity of chloride homeostasis in sensory reticular nucleus regions may reflect functional adaptations that facilitate activity-dependent plasticity during critical periods for sensory circuit refinement, but it simultaneously creates windows of vulnerability when metabolic stress disrupts the delicate balance between chloride uptake and extrusion.

### 3.3 Developmental emergence of reticulothalamic circuits

**Figure 2:**
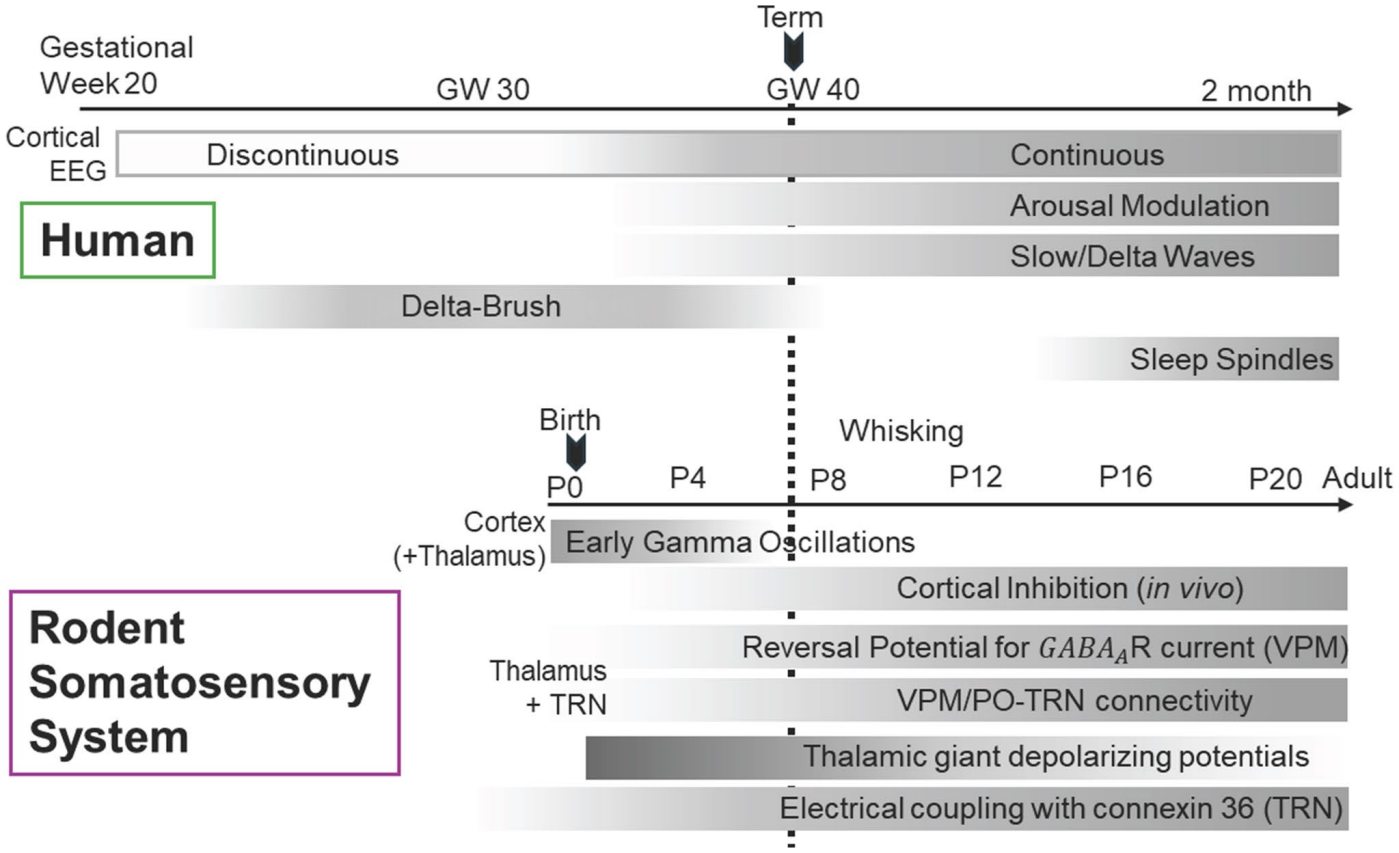
Development of thalamocortical system. Aligned developmental timelines of human **(top)** and rodent **(bottom)** thalamocortical (TC) circuits, matched at human birth with rat postnatal day 7 (P7). The schematic summarizes sequential maturation of cortical oscillations and inhibitory mechanisms within the thalamo-reticular pathways, which mature asynchronously across sensory systems (adapted from Murata and Colonnese, 2019). Feedforward and feedback connections between the ventral posterior nucleus (VPM) and TRN reach functional maturity around P6–P7, whereas visual (LGN–TRN) and higher-order pathways develop later (not shown). This temporal gradient defines a window of circuit reorganization marked by heightened excitability and metabolic demand, rendering ventral TRN and its early-maturing thalamic partners particularly susceptible to hypoxic–ischemic injury in the neonatal and pediatric brain. In rodents, electrical coupling among TRN neurons is already widespread at birth (60–80%) but strengthens markedly during the second postnatal week, coinciding with the rise in connexin-36–mediated conductance and decline in membrane resistance. In contrast, chemical (GABAergic) synapses within TRN peak early and regress after P14, leaving mature TRN networks dominated by electrical rather than chemical intra-reticular connectivity. Transient excitatory GABA signaling during this period drives thalamic giant depolarizing potentials (tGDPs), a spontaneous rhythmic discharge that is characteristic of immature oscillatory networks, at the first postnatal week. This developmental transition from mixed chemical–electrical coupling to primarily electrical coordination defines a critical window of heightened network excitability and metabolic demand, rendering the ventral TRN and its thalamocortical partners especially susceptible to hypoxic–ischemic injury.

Studies of anatomical development of thalamic relay nuclei and the TRN demonstrate their reciprocal connectivity follows distinct temporal trajectories across sensory systems. In the somatosensory pathway, both excitatory VPM-to-TRN and inhibitory TRN-to-VPM synapses are already functional at birth (P1) and undergo a rapid, coordinated strengthening around postnatal days 6 to 7, when synaptic conductance increases fourfold and connection reliability reaches near-adult levels (Evrard and Ropert, 2009). In contrast, the visual circuit develops sequentially: inhibitory feedback from TRN to dLGN is established during the first postnatal week (P2–P7) and matures by week 3, whereas excitatory feedforward input from dLGN to TRN emerges later, with functional synapses appearing after P10 and reaching full maturity only by postnatal day 21 (Figure 2) (Campbell et al., 2024). This asynchronous pattern highlights a system-specific developmental strategy in which somatosensory circuits achieve early reciprocal integration, while visual circuits rely on a delayed, stepwise assembly.

During the first two postnatal weeks, GABAergic connections among TRN neurons are common and drive spontaneous thalamic giant depolarizing potentials (tGDP) that peak around postnatal day 7 and disappear by the 3rd postnatal week (Pangratz-Fuehrer et al., 2007). The depolarizations are abolished by GABA receptor antagonism, persist when ionotropic glutamate receptors are blocked, and are potentiated by benzodiazepines that enhance alpha5-containing GABA receptors transiently expressed during this developmental window. The ionic mechanisms underlying giant depolarizing potentials reflect the unique physiological state of the immature TRN creates periodic depolarizations that enhance calcium entry through voltage-gated channels and low-threshold T-type channels, trigger rebound bursts in connected relay neurons, and drive spindle-burst-like patterns in immature sensory cortices (Akerman and Cline, 2006; Cancedda et al., 2007; Rheims et al., 2008; Wang and Kriegstein, 2008). Perforated-patch recordings confirm depolarizing GABA reversal potential, indicating that tGDP occurs in the context of elevated intracellular chloride and depolarizing GABA responses. Concurrent gap junction coupling provides low-resistance pathways for spatial spread, enabling synchronous depolarization across large reticular nucleus territories. In humans, developmental EEG features in the preterm infants exhibit delta-brushes, bursts of fast activity superimposed on slow waves, that likely reflect thalamus-driven synchrony analogous to rodent spindle-bursts occurring when GABAergic signaling retains depolarizing properties in parts of the thalamocortical system.

Functionally, tGDP represents more than developmental epiphenomena; it provides structured periodic inputs essential for normal thalamocortical development. The rhythmic depolarizations entrain cortical activity patterns, promote activity-dependent competition among thalamocortical axons targeting common cortical territories, and establish the foundation for later spindle oscillations. As corticothalamic drive strengthens and KCC2-mediated chloride extrusion matures in relay nuclei, the network transitions from tGDP-dominated synchrony to spindle-capable circuits, with the TRN switching roles from depolarizing organizer to hyperpolarizing gate (An et al., 2014; Tiriac and Blumberg, 2016; Zolnik and Connors, 2016). This developmental transition can be directly observed in the changing characteristics of thalamocortical oscillations, with early slow coordinated bursts gradually transforming into faster, more state-dependent rhythms as chloride homeostasis mature (Khazipov et al., 2004; Luhmann and Prince, 1991; Minlebaev et al., 2007; Yang et al., 2016). Similarly, around term infants and into early postnatal months, quiet-sleep trace becomes increasingly state-dependent, gradual emergence of canonical sleep spindles, signaling maturation of reticular nucleus-mediated hyperpolarizing inhibition and corticothalamic modulation. The developmental trajectory of thalamocortical EEG strongly implies a giant depolarizing potential to spindle maturation sequence in which chloride homeostasis and reticulothalamic specialization occupy central roles.

### 3.4 Thalamoreticular pathway is topographic and cell-type specific

**Figure 3:**
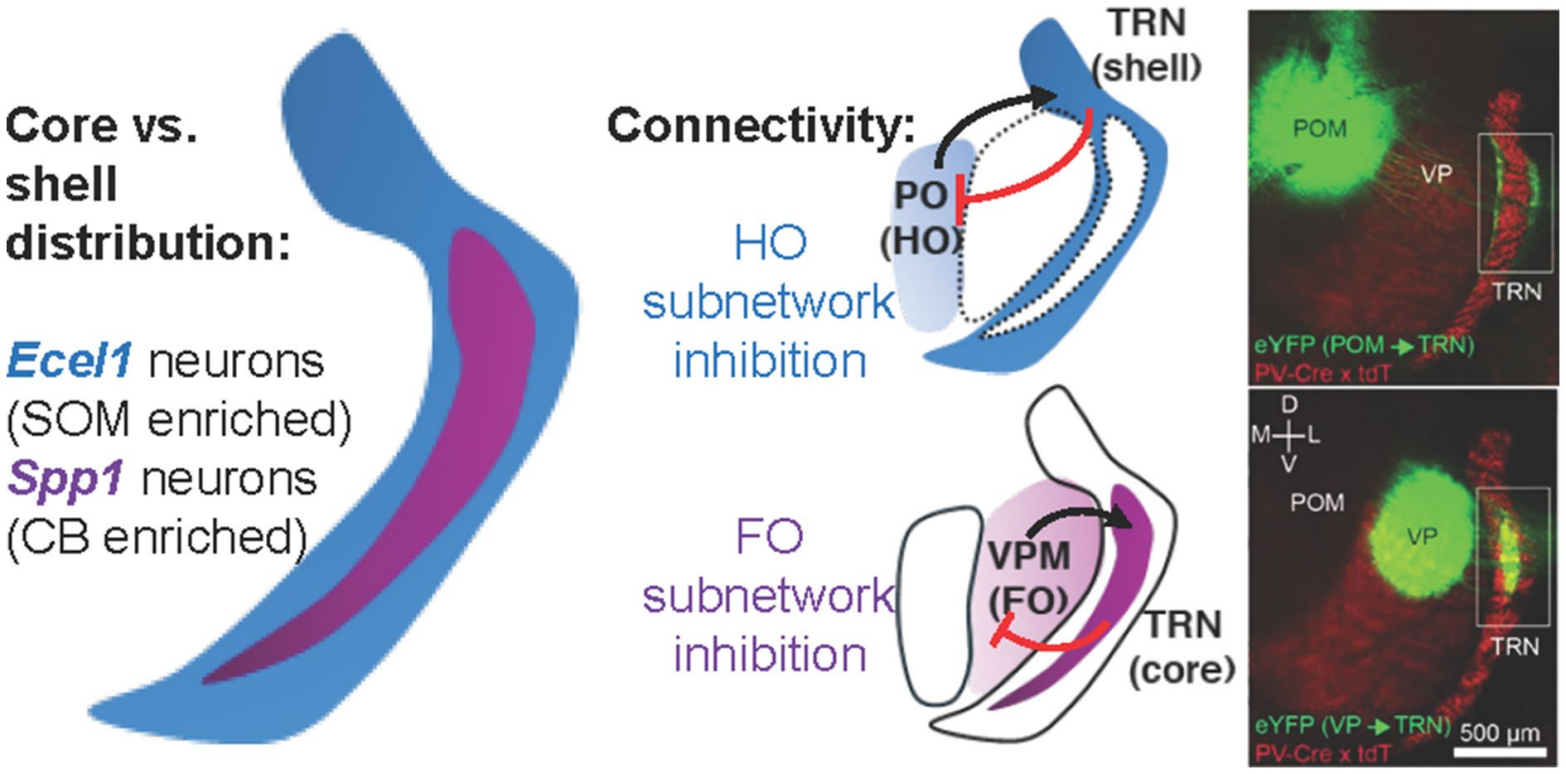
Thalamoreticular pathway is topographic and cell-type specific. Schematic illustration of the inhibitory reticulothalamic system showing distinct subnetworks connecting the TRN with first-order (FO) and higher-order (HO) thalamic nuclei. *Spp1*-expressing (*Spp1^+^*) TRN neurons occupy the central core of the TRN, exhibit high-frequency burst firing, and project predominantly to FO nuclei such as the ventral posterior (VPM/VPL). *Ecel1*-expressing (*Ecel1^+^*) neurons localize to the surrounding shell, display lower burst propensity, and project to HO nuclei including the posteromedial (PO) and mediodorsal (MD) (not shown) thalamus, highlighting parallel TRN subnetworks with distinct input–output relationships and cortical convergence patterns (immunofluorescence micrograph adapted from Martinez-Garcia et al., 2020). Functionally, these subnetworks form topographically organized inhibitory loops that differentially modulate sensory and associative thalamic activity. The preferential early maturation and higher intrinsic excitability of the *Spp1^+^*–FO network may contribute to its selective vulnerability during neonatal and pediatric ischemic stress, when metabolically demanding burst-synchronizing circuits are most active.

By the end of the developmental processes described above, *adult* reticulothalamic architecture reflects a finely stratified organization of reciprocal inhibitory–excitatory loops linking TRN neurons to first-order (FO) and higher-order (HO) thalamic nuclei. This mature topographic organization emerges from the sequential assembly of feedforward and feedback connections outlined in *Section 3.3*. FO nuclei such as the VPM, ventral anterior (VA), and ventral lateral (VL) nuclei receive focal inhibitory projections from core regions of the TRN (Figure 3) (Diamond et al., 2008; Lam and Sherman, 2011; Li et al., 2020; Martinez-Garcia et al., 2020). These connections are dominated by parvalbumin- and calbindin-expressing neurons that confer temporally precise inhibition suited to discrete sensory and motor signals (Conley et al., 1991; Fishell and Rudy, 2011; Vaughn et al., 2024). In contrast, HO nuclei such as the posterior medial and central lateral nuclei receive broader and more diffuse inhibitory input from somatostatin-expressing neurons located along the outer shell of the TRN (Clemente-Perez et al., 2017; Graybiel and Elde, 1983; Vaughn et al., 2024). This topographic division corresponds to modality-specific sectors: dorsal regions link to visual nuclei, ventral to auditory, medial to somatosensory, and anterior to motor and limbic systems (Guillery and Harting, 2003; Pinault, 2004; Sherman, 2016).

The spatial segregation of inhibitory subtypes reflects functional specialization. Parvalbumin (PV) neurons form the dominant population and exhibit intrinsic rhythmicity, mediating rapid, phasic inhibition critical for the generation of spindles and sleep-related oscillations (Fogerson and Huguenard, 2016; Halassa et al., 2014). In the somatosensory sector of the TRN, a significant subset of this population (~48%) at the central core area co-expresses both PV and Calbindin (CB), which contributes to the precision of FO thalamic relay control (Martinez-Garcia et al., 2020; Vaughn et al., 2024). Of particular relevance are the findings that CB has been used in several neuropathological studies, which have explored the possible relation between CB and neuronal protection (Kishimoto et al., 1998; Shetty and Turner, 1998), as well as that the CB immunoreactivity is practically limited to the dorsal thalamus during ontogenesis (Contreras-Rodríguez et al., 2003; Enderlin et al., 1987; Puelles et al., 1992). Somatostatin (SOM) neurons (~64%), localized to the dorsal and lateral margins, preferentially interact with HO thalamic nuclei and regulate state-dependent modulation of cortical synchrony (Clemente-Perez et al., 2017; Martinez-Garcia et al., 2020; Vaughn et al., 2024). Electrical coupling between neighboring TRN neurons through connexin-36–mediated gap junctions further enhances synchrony and facilitates coordinated inhibition across functional sectors (Condorelli et al., 2000; Haas et al., 2011; Landisman et al., 2002). Recent single-cell RNA sequencing analysis and genetic mouse line approaches not only confirm the heterogeneity of TRN GABAergic neurons, but also identify that CB subpopulation overlap with secreted phosphoprotein 1 (*Spp1*) subnetwork neurons (Hartley et al., 2024; Li et al., 2020). *Spp1 +* TRN neurons feature lower average input resistance, more excitable, larger AP amplitude, bigger in average cell size, and express high levels of metabotropic glutamate receptor 3 (mGluR3) (Hartley et al., 2024; Li et al., 2020). Collectively, these findings define the TRN as a modular, topographically ordered network that differentially modulates thalamocortical information streams according to sensory modality and behavioral context.

The cell-type specialized, topographic precise *adult* thalamoreticular pathways develop gradually across distinct postnatal windows (Figure 2). In rodents, PV and CB expression within core TRN neurons emerges during the first postnatal week and strengthens through P14, coinciding with the maturation of reciprocal VPM-TRN connectivity (see *Section 3.3*). SOM expression in shell neurons follows a delayed trajectory, reaching adult patterns only after P21. This asynchronous maturation creates transient developmental states where ventral sensory sectors exhibit adult-like connectivity and excitability while still retaining immature chloride homeostasis and elevated electrical coupling, as directly measured in slice recordings (Klein et al., 2018; Schmidt et al., 2018). Whether this temporal mismatch between structural maturation and physiological refinement similarly defines vulnerability windows in brain exposed to pediatric asphyxial event remains to be determined, though the selective injury of sensory TRN sectors observed in pediatric cardiac arrest models (Ross and Duhaime, 1989; Ton et al., 2021) is consistent with susceptibility of early-developing FO-linked pattern.

## 4 Mechanisms of selective vulnerability: why the thalamic reticular nucleus fails under hypoxic–ischemic stress

### 4.1 Chloride dysregulation and the developmental liability of immature KCC2 expression

**Figure 4:**
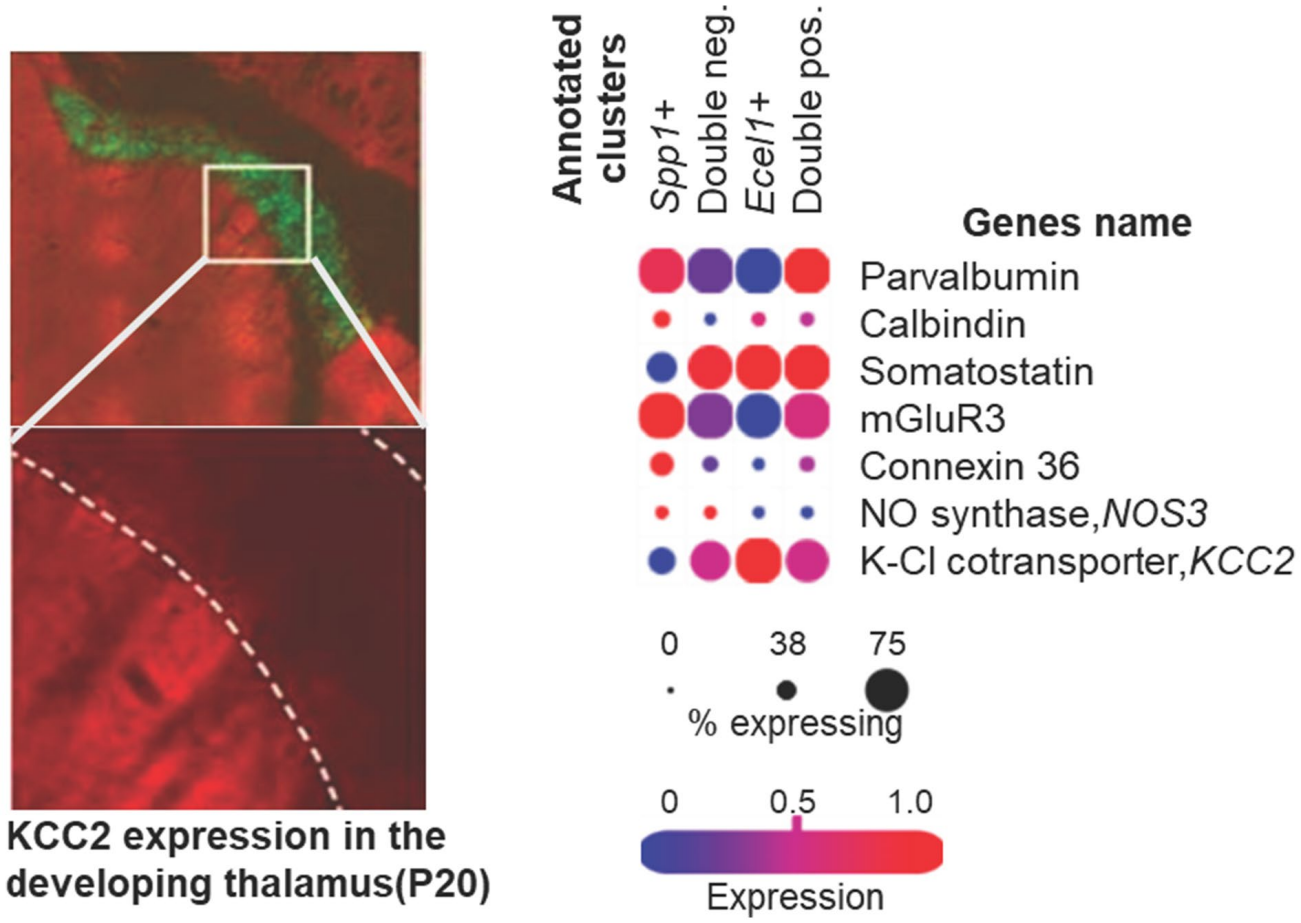
Cellular organization of TRN. Left: Representative immunostaining images show differential KCC2 and NKCC1 expression ventrobasal thalamus (VB) versus TRN in developing mouse thalamus at P20. KCC2 (green) is strongly expressed at somata and dendrites of VB neurons, whereas KCC2 immunoreactivity is virtually absent in the TRN. MAP2 (red) serves as a somatodendritic marker (adapted from Schmidt et al., 2018). Right: Dot plot illustrating transcriptomic markers (*Spp1*, *Ecel1*) used to define TRN cell clusters (raw data from GSE145273, Li et al., 2020). The dot size represents the proportion of cells expressing each gene, and color intensity indicates average expression of key functional markers, including parvalbumin, calbindin, somatostatin, mGluR3, connexin-36, and KCC2.

Developmental immunohistochemistry confirms that KCC2 expression remains persistently low in the TRN relative to ventrobasal thalamus from P5 through P40, with the canonical postnatal upregulation of KCC2 markedly blunted in the ventral part of TRN (Figure 4). Throughout postnatal development, the anterior and lateral segments acquire modestly elevated KCC2 immunoreactivity (approximately 120–134% of the TRN mean), whereas the central somatosensory segments retain the lowest expression (approximately 79–82% of the TRN mean) (Klein et al., 2018). Functional recordings confirm that this anatomical pattern translates to physiological differences, with TRN neurons exhibiting a more depolarized GABA reversal potential (approximately −62 mV) compared to relay neurons (approximately −79 mV), and with greater variability in chloride regulation among TRN neurons than among their relay counterparts (Klein et al., 2018). Notably, Schmidt et al. (2018) demonstrated that KCC2 protein in adult TRN reaches only 9% of the level observed in ventrobasal thalamus, confirming that the developmental deficit persists into maturity (Schmidt et al., 2018).

The prolonged immaturity of chloride homeostasis in sensory TRN sectors may reflect functional adaptations that facilitate activity-dependent plasticity during critical periods for sensory circuit refinement, but it simultaneously creates windows of vulnerability under metabolic stress. During early postnatal activity, network events such as tGDPs drive recurrent GABA release that can transiently raise intracellular chloride and depolarize TRN neurons. When oxygen and glucose are depleted, ATP loss suppresses Na^+^/K^+^-ATPase function, disrupting ionic gradients that sustain chloride transport. NKCC1-mediated influx combined with impaired KCC2 extrusion accelerates chloride accumulation, further depolarizing the GABA reversal potential. As a result, TRN neurons operate near the depolarizing range of GABA even at baseline, and modest increases in intracellular chloride readily shift the GABA reversal potential toward depolarization, significantly contributes to the mechanisms of excitotoxicity and associated neuronal loss (Jaenisch et al., 2010; Pellegrino et al., 2011; Raimondo et al., 2017; Van Damme et al., 2003).

### 4.2 Role of neuronal electrical synapses in cell death during hypoxic–ischemic injury

The immature TRN expresses high levels of Cx36, forming dense electrical synapses that coordinate activity across reticular ensembles during early development (Landisman et al., 2002; Pangratz-Fuehrer et al., 2007). Cx36-containing electrical synapses remain particularly abundant in ventral sensory sectors where chloride extrusion is weak and GABA signaling remains depolarizing through the early postnatal period. The coexistence of high Cx36 expression and immature chloride regulation in the developing TRN is hypothesized to facilitate the propagation of ischemic depolarization and calcium influx.

**Figure 5:**
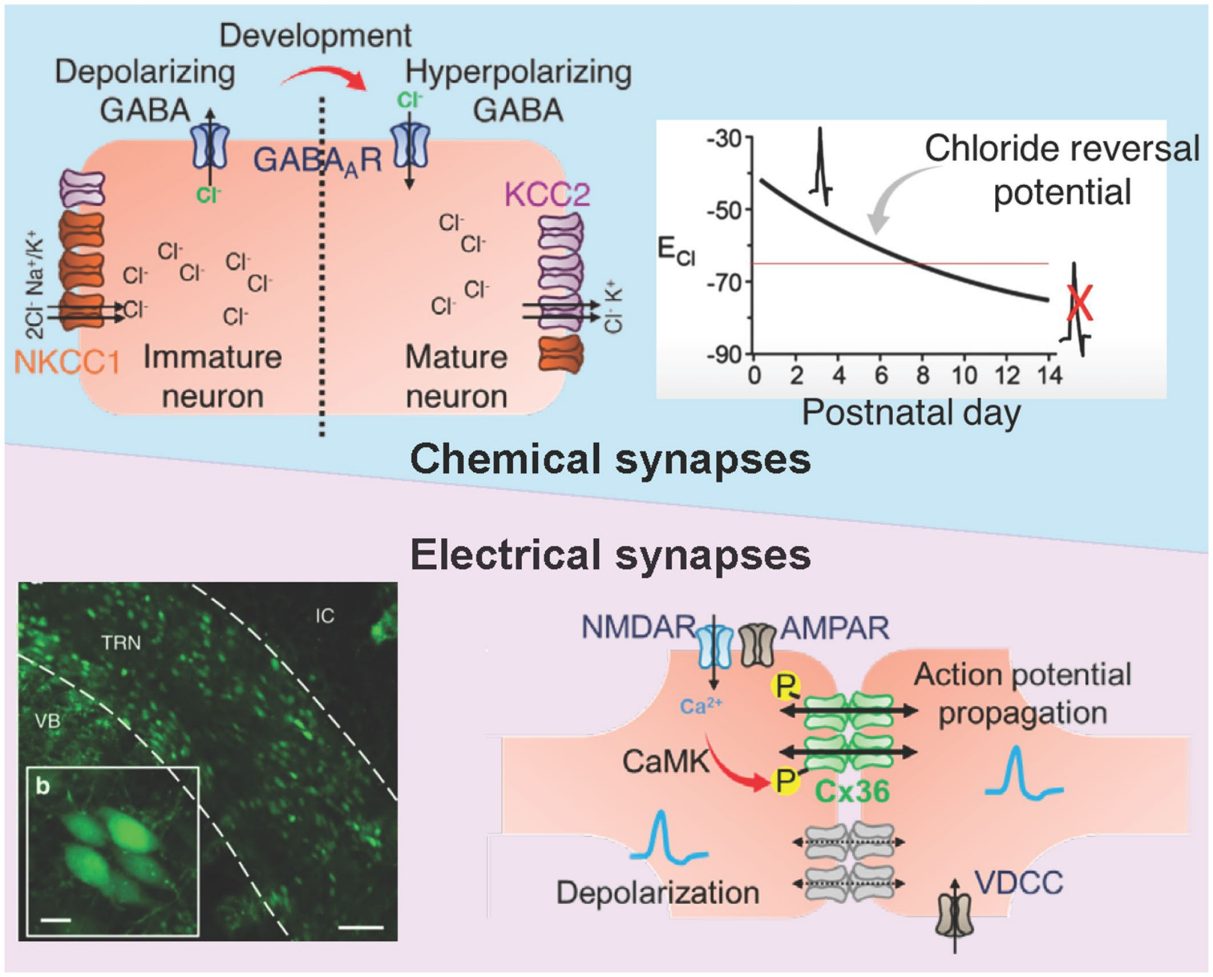
Development of intra-TRN connectivity. Schematic on the top representation of the developmental shift in GABAergic signaling polarity within the TRN. During early postnatal stages, high intracellular chloride maintained by dominant NKCC1 activity renders GABA depolarizing, supporting excitatory synchronization and tGDPs. As KCC2 expression increases through late infancy, chloride extrusion strengthens, converting GABA action to inhibitory and stabilizing network oscillations. Disruption of this chloride gradient during hypoxia-ischemia prolongs the depolarizing phase, enhancing excitotoxic drive and network hypersynchrony. The bottom panel contains fluorescence images from Gjd2-EGFP transgenic mice illustrating extensive expression of connexin-36–driven EGFP in TRN neurons [VB, ventrobasal nucleus; IC, internal capsule; scale bars = 100 μm adapted from Palacios-Prado et al. (2014)]. The accompanying cartoon depicts electrical coupling between TRN neurons mediated by connexin-36 gap junctions, highlighting calcium influx through voltage-dependent calcium channels (VDCCs) and NMDA receptors during synchronous depolarization. This configuration promotes high-frequency network oscillations but also facilitates propagation of depolarizing calcium loads during metabolic stress. Together, these features define a developmental window of heightened TRN vulnerability, when excitatory GABA signaling and dense electrical coupling converge to amplify ischemic injury in the neonatal and pediatric brain.

During hypoxic–ischemic events, excessive glutamate release activates group II metabotropic glutamate receptors which trigger a cAMP/PKA-dependent cascade that increases both Cx36 expression and electrical coupling within hours (Figure 5) (Wang et al., 2012; Wang et al., 2015). This injury-induced upregulation occurs in parallel with NMDA receptor hyperactivation and intracellular calcium overload and excitotoxic injury (de Rivero Vaccari et al., 2007; Frantseva et al., 2002). Experimental models of ischemia, traumatic brain injury, and epilepsy consistently demonstrate that pharmacologic blockade or genetic deletion of Cx36 markedly reduces neuronal death, underscoring its causal role in injury propagation (Cho et al., 2024). Conversely, sustained activation of mGluR-II augments developmental increases in electrical coupling and enhances NMDA-mediated neurotoxicity, while its inhibition prevents both coupling upregulation and excitotoxic death (Wang et al., 2012). Together, these findings support a framework in which Cx36-mediated electrical synapses in the immature TRN act as active drivers of ischemic injury cascades rather than passive conduits.

### 4.3 Circuit-level vulnerability of the immature thalamic reticular nucleus to first-order thalamic input during hypoxic–ischemic injury

The thalamoreticular projection is most prevalent in the immature TRN because corticoreticular input develops later in postnatal life. Anatomically, this early thalamic–reticular drive originates from FO sensory thalamic nuclei, providing excitatory glutamatergic input to modality-specific TRN sectors (Guillery et al., 1998; López-Bendito and Molnár, 2003; Molnar et al., 1998; Pinault, 2004). Developmental studies demonstrate that thalamoreticular circuits do not mature synchronously across sensory modalities (see *section 3.3*). This asynchronous assembly means that, during the developmental window approximately the late fetal through early post-infant period in humans, spanning the last trimester of gestation to about 2–4 years of age, the somatosensory (VPM–ventral TRN) network is already fully engaged in reciprocal excitation–inhibition loops, while the visual (dLGN–dorsal TRN) network remains functionally immature. The early maturation of the VPM–ventral TRN pathway positions these neurons to receive robust glutamatergic afferets comparing to dorsal TRN. During ischemia, glutamate release through reversed transporter operation drives neuronal depolarization (Choi and Rothman, 1990; Johnston et al., 2001; Rossi et al., 2000). We hypothesize that ventral TRN neurons, receiving this excitatory drive through their early-maturing afferents while retaining immature chloride extrusion capacity, become selectively vulnerable to the degeneration documented in human cases and animal models of asphyxial injury.

The immature TRN operates within a constrained physiological framework that shapes its selective vulnerability to hypoxic–ischemic stress which cannot be attributed solely to their GABAergic phenotype, as cortical GABAergic interneurons and anterior TRN neurons were preserved under identical ischemic conditions. Ton and Shoykhet therefore proposed that intrinsic differences in calcium buffering capacity, metabolic load, or excitatory drive may contribute to this topographic selectivity (Ton et al., 2021; Welsh et al., 2002). Extending this interpretation, the limited chloride extrusion capacity of ventral TRN neurons, due to sparse KCC2 expression, together with extensive Cx36–mediated electrical coupling and strong excitatory thalamic inputs, further amplifies the susceptibility of the posterior ventral sector to ischemic stress (Figure 5). Consequently, the abnormal electrophysiological patterns observed after perinatal asphyxia, such as burst suppression, loss of sleep–wake cycling, and reduced spindle activity, likely reflect disrupted developmental assembly within these inhibitory networks.

### 4.4 Alternative mechanisms of regional susceptibility

To contextualize the proposed developmental-physiological model, it is necessary to examine the consistency of subcortical vulnerability across diverse experimental and clinical paradigms. The selective susceptibility of the TRN—and its resulting functional consequences—has been documented across a variety of species. Table 1 provides a comprehensive comparative summary of these findings, illustrating the neuroanatomical and mechanistic commonalities that persist across different maturation states and resuscitation strategies, anchored by foundational human autopsy data.

**Table 1:** Comparative summary of post-cardiac arrest brain injury literature cited in the current review.

Species/Model	Method of ROSC	Duration of No-flow/Insult	Therapeutic hypothermia (Y/N)	Neurological assessment timepoints	Summary of neuropathology	Main citations
Human (Clinical)	Clinical Resuscitation	Various (approx. 5–15 + min)	N (Standard care at time)	Autopsy (survival: 14 h to 10 weeks)	Selective RT loss in prefrontal-associated sectors after short arrests; sparing of RT in long arrests (>10 min) involving relay nuclei death.	Ross and Graham (1993)
Rat (Adult)	Resuscitation from CA	10 min Cardiac Arrest	N	7 days post-ROSC	50–60% reduction in RT neuronal density; protection achievable via AMPA antagonist (NBQX) administered post-ROSC.	Ross and Graham (1993)
Rat (P17 developing)	Asphyxial CA and CPR	9 min Asphyxial CA	N	48 h to 8 weeks post-ROSC	Chronic hyperactivity and disorganized receptive fields in thalamocortical circuits; RT microglial activation.	Shoykhet et al. (2012) and Middleton et al. (2017)
Lamb (Near-term)	CPR and Ventilation	5 min Asystole (via UCO)	N	Daily for 6 days	Injury to hippocampus and cingulate cortex; significant loss of O4 + oligodendrocytes and reactive gliosis.	Mike et al. (2022, 2023)
Piglet (4-week-old)	ECPR	30 or 60 min prolonged CPR	N	22–24 h post-ROSC	Duration-dependent mitochondrial respiration (OXPHOS) failure; early metabolic markers of cerebral injury.	Slovis et al. (2023)
Piglet (Newborn)	Stabilization	10–25 min Hypoxia (8% O₂)	N	48 h post-insult	Exacerbated cell death in internal capsule and sensorimotor cortex.	Martinello et al. (2019)

CA, cardiac arrest; CPR, cardiopulmonary resuscitation; ECPR, extracorporeal cardiopulmonary resuscitation (resuscitation using a heart-lung machine); ROSC, return of spontaneous circulation; UCO, umbilical cord occlusion; TH, therapeutic hypothermia; AMPA, α-amino-3-hydroxy-5-methyl-4-isoxazolepropionate (a type of glutamate receptor); NBQX, a specific pharmacological antagonist used to block AMPA receptors; OXPHOS, oxidative phosphorylation (mitochondrial energy production); TUNEL, terminal deoxynucleotidyl transferase dUTP nick end labeling (a method for detecting apoptotic DNA fragmentation/cell death).

While the developmental convergence of chloride dysregulation and electrical coupling offers a robust framework for TRN vulnerability, other intrinsic and extrinsic factors likely contribute to the observed topographic selectivity. Regional variations in vascular supply represent a primary extrinsic consideration, as the ventral TRN is predominantly served by terminal branches of the thalamogeniculate arteries. These vessels may possess distinct autoregulatory capacities or perfusion pressures compared to the thalamoperforating branches supplying the anterior TRN, potentially rendering the sensory sectors more sensitive to hemodynamic fluctuations during cardiac arrest or systemic hypotension (Tierradentro-Garcia et al., 2023). Furthermore, the intrinsic metabolic profile of TRN neurons, marked by high-frequency bursting and rapid repolarization (Cox et al., 1997), imposes high ATP demands that may lower the threshold for cellular failure during hypoxic–ischemic energy depletion (Ton et al., 2021; Welsh et al., 2002). This metabolic susceptibility may be compounded by regional differences in mitochondrial resilience and calcium buffering capacity, such as varying expression levels of calbindin or differences in mitochondrial density across TRN segments. A reduced capacity for calcium sequestration or oxidative stress management could accelerate the transition from reversible cellular stress to irreversible cell death, acting either independently of or in synergy with synaptic-level dysregulation. Clarifying the relative contributions of these alternative mechanisms remains an important area for future investigation to fully characterize the landscape of subcortical injury.

## 5 Translational opportunities and therapeutic directions

### 5.1 Current neuroprotective strategies and their limitations

Therapeutic hypothermia remains the only proven neuroprotective intervention for neonatal hypoxic–ischemic encephalopathy, yet its efficacy is modest with approximately half of treated infants still experiencing death or major disability (Azzopardi, Denis V. et al., 2009; Jacobs et al., 2007; Shankaran et al., 2005). The mechanisms underlying hypothermia’s neuroprotective effects remain incompletely understood but likely involve global reductions in metabolic rate, decreased excitotoxic neurotransmitter release, and attenuation of inflammatory cascades (Mallard et al., 2014; Nakashima et al., 1995; Thoresen et al., 1997; Wassink et al., 2014). Notably, therapeutic hypothermia shows differential efficacy across brain regions and injury types, with some evidence suggesting better protection of cortical compared to deep gray matter structures (Laptook et al., 2001; Roelfsema et al., 2004). The persistent vulnerability of thalamic structures including reticular nucleus despite hypothermia treatment suggests that mechanisms specific to these regions may require targeted interventions beyond global metabolic suppression.

The failure of hypothermia to consistently benefit pediatric cardiac arrest patients, despite efficacy in neonatal hypoxic–ischemic encephalopathy, underscores important differences between injury contexts and developmental stages (Gunn et al., 2017). Cardiac arrest in older children produces different patterns of regional vulnerability and may engage distinct pathophysiological mechanisms compared to perinatal asphyxia (Moler et al., 2015; Moler et al., 2017). The selective thalamic injury observed in pediatric cardiac arrest models, with prominent reticular nucleus involvement, may reflect these mechanistic differences (Ross and Graham, 1993; Shoykhet et al., 2012; Ton et al., 2021). Understanding why certain vulnerable populations and injury patterns respond poorly to current neuroprotective strategies is essential for developing next-generation interventions that address inadequacies of existing approaches.

The translation of chloride homeostasis and connexin-targeted therapies from experimental models to clinical practice faces substantial challenges that must be carefully considered. First, the developmental trajectories of KCC2 and NKCC1 expression differ markedly between rodents and humans, with human cortical maturation extending over years rather than weeks, potentially altering both the therapeutic window and dosing requirements for agents like bumetanide or CLP290 (see Section 5.2). Second, bumetanide exhibits poor blood–brain barrier penetration in its current formulation, achieving only 0.3% cerebrospinal fluid concentration relative to plasma levels (Dzhala et al., 2024), which may explain inconsistent clinical outcomes despite promising preclinical results. Third, connexin-blocking peptides such as C1B1 or connexin-43 mimetic peptides demonstrate short half-lives and limited central nervous system bioavailability, requiring repeated administration or novel delivery strategies such as nanoparticle carriers or modified peptide scaffolds to achieve sustained therapeutic concentrations during the critical 24 to 72 h post-injury window. Fourth, the cell-type specificity of connexin expression introduces both opportunity and complexity, as connexins expression across broad range of cells, including neuronal populations, astrocytes, and oligodendrocytes. These considerations underscore the need for iterative refinement of delivery methods, age-specific pharmacokinetic studies, and comprehensive safety evaluations in large animal models that more closely approximate human neurodevelopment before advancing chloride- or connexin-targeted interventions to clinical trials in neonates and children.

### 5.2 Targeting chloride homeostasis as a therapeutic strategy

Disrupted chloride homeostasis is a critical determinant of neuronal excitability and vulnerability within the thalamic reticular nucleus after hypoxic–ischemic injury. Bumetanide is widely used to inhibit NKCC1 in both experimental and clinical studies, which limits chloride influx and restores the hyperpolarizing action of GABA (Ben-Ari et al., 1989; Ben-Ari et al., 2007; Javaheri et al., 1993; Kharod et al., 2019; Löscher and Kaila, 2022). However, evidence from recent experimental studies indicates that the efficacy of bumetanide is highly dependent on both timing and combinatorial context (Dzhala et al., 2005; Erker et al., 2016; Töllner et al., 2014). In neonatal hippocampal and thalamic models of oxygen–glucose deprivation, early administration of bumetanide reduces neuronal [Cl^−^]_i_ accumulation and partially restores GABAergic inhibition, but its effectiveness declines rapidly as excitotoxicity and cytotoxic edema progress (Ding et al., 2016; Löscher and Kaila, 2021). Recently, Dzhala et al. (2024) demonstrated that combining bumetanide with phenobarbital suppresses ictal-like discharges even when treatment is delayed up to 10 h after injury, whereas either drug alone shows diminished benefit at later stages, suggesting a synergistic mechanism that stabilizes chloride equilibrium across a wider therapeutic window.

Beyond NKCC1 inhibition, recent approaches target restoration of KCC2-mediated chloride extrusion. In neonatal ischemic seizure models, selective enhancement of KCC2 function using CLP290 or its parent compound CLP257 reinstates hyperpolarizing GABAergic signaling and rescues phenobarbital-resistant seizures (Gagnon et al., 2013). Sullivan et al. (2021) showed that CLP290 prevents both acute seizure recurrence and long-term epileptogenesis by post-translational modification at phospho-Ser(940) to increase the cell surface stability of KCC2 (Sullivan et al., 2021). Similarly, Carter et al. (2018) found that inhibiting TrkB signaling with ANA12 reversed ischemia-induced KCC2 downregulation and restored phenobarbital sensitivity (Carter et al., 2018; Cazorla et al., 2011), further confirming that post-ischemic KCC2 hypofunction is a key molecular driver of disinhibition. Together, these studies demonstrate that pharmacological enhancement of KCC2 function, either directly (CLP290) or indirectly through modulation of TrkB–BDNF signaling (ANA12), can normalize chloride gradients and improve both acute and long-term outcomes after neonatal hypoxic–ischemic injury.

### 5.3 Neuroprotective role of connexin blockade in neonatal hypoxic–ischemic injury and pediatric cardiac arrest

Connexin blockade represents a different neuroprotective strategy than therapeutic hypothermia, which acts systemically to suppress global metabolic activity. Connexin-based interventions exploit the restricted anatomical distribution and cell-type-specific expression patterns of neuronal gap junction proteins to selectively interrupt injury propagation in vulnerable circuits (Belousov and Fontes, 2013; Davidson et al., 2015). A growing body of experimental work indicates that connexin-based electrical synapses play an active role in the amplification of hypoxic–ischemic injury in the immature brain. In addition, neonatal asphyxia and pediatric cardiac arrest induce rapid upregulation of neuronal connexins (Davidson et al., 2012a; Davidson et al., 2012b; Li et al., 2015; Wang et al., 2014), paralleled by opening of hemichannels in glia and endothelium, which further increases extracellular ATP, glutamate, and calcium flux (Gomes et al., 2005; Takeuchi et al., 2006; Ye et al., 2003). The injury-triggered opening of these large-pore channels occurs through multiple mechanisms. During energy depletion, loss of the ATP-dependent membrane potential gradient and reduced extracellular calcium promote hemichannel opening in both neurons and glia (Contreras et al., 2002; Orellana et al., 2010). Additionally, reactive oxygen species and nitric oxide, which accumulate during hypoxia-ischemia, directly enhance channel permeability through post-translational modifications of connexin proteins (Lillo et al., 2019; Retamal et al., 2006). These injury-associated connexin responses suggest that targeted blockade might interrupt the spread of secondary injury during the latent and early secondary phases of post-asphyxial degeneration (Davidson et al., 2018; Zhou et al., 2019).

Experimental model shows that deleting neuronal Cx36 prevents injury-induced coupling increases, calcium overload, and NMDA excitotoxicity in oxygen–glucose deprivation and stroke models (de Rivero Vaccari et al., 2007; Fontes et al., 2015; Ma et al., 2018). Parallel findings emerge from double connexin knockouts involving pannexins: mice lacking Px1 and Px2 exhibit smaller infarcts and better functional recovery after middle cerebral artery occlusion compared with wild-type animals (Bargiotas et al., 2011). The temporal pattern of channel opening differs between connexins and pannexins during ischemic injury. Cell culture studies demonstrate that ATP release during the initial ischemic period occurs through both pannexin channels (approximately one third) and connexin hemichannels (approximately two thirds), but during reperfusion, ATP efflux becomes exclusively dependent on connexin hemichannels (Kim et al., 2017). This temporal shift reflects the self-regulatory properties of pannexin channels, which close in response to elevated extracellular ATP concentrations through a negative feedback mechanism, whereas connexin hemichannels remain open and continue to release ATP, perpetuating the injury cascade (Iwabuchi and Kawahara, 2011; Qiu and Dahl, 2009). Therefore, these *in vitro* data identify connexin- and pannexin-mediated membrane permeabilization as mediators of secondary injury cascades.

Pharmacologic blockade, such as synthetic peptides that are designed to inhibit connexin channel function, demonstrates neuroprotective actions in various brain injury models (Abudara et al., 2014; O’Carroll et al., 2008; Wang et al., 2013). The connexin-blocking peptide C1B1 reduces connexin-mediated conductance and protects against cell death in models of oxidative and metabolic stress by suppressing hemichannel opening through extracellular loop sequence interactions (Lin and Takemoto, 2007). C1B1 works by competitively binding to 14–3-3 docking proteins, which releases endogenous PKCγ from its inactive cytosolic complex. The freed PKCγ then phosphorylates connexin proteins to close the channels. This mechanism prevents apoptotic signals from spreading between cells, blocking the “bystander effect” where death signals propagate from injured to healthy adjacent cells through open gap junctions and hemichannels. Similar results occur with peptide inhibitors targeting connexin 43 (Cx43) hemichannels improve neuronal, oligodendrocyte, and interneuron survival when delivered within hours after global ischemia in near-term fetal sheep (Davidson, et al., 2013a,b; Davidson et al., 2012b; Galinsky et al., 2017), a highly comparable model for neonatal and fetal ischemia (Gunn et al., 1997). Although Cx43 is expressed primarily in glia and endothelium rather than TRN neurons (Dermietzel et al., 2000; Nagy et al., 2003; Davidson et al., 2013b), hemichannel opening in these compartments participates in the pathological cascade that amplifies injury after hypoxia–ischemia. The cell-type segregation of connexin expression creates distinct but converging injury pathways and explain why combined blockade of multiple connexin isoforms may provide superior neuroprotection compared to targeting a single connexin species.

Together, these mechanistic and preclinical findings support a model in which connexin and pannexin channels operate as amplifiers of post-asphyxial injury. In the developing TRN, high baseline electrical synaptic coupling through Cx36 creates a vulnerable substrate that potentiates hypoxic depolarization and synchronized excitotoxic waves, following by secondary degeneration resulting from widespread opening of Cx43 and pannexin hemichannels in glia and endothelium drives ATP release (Baroja-Mazo et al., 2013; Stout et al., 2002) and persistent inflammation (He et al., 2016; Omer et al., 2021). The convergence of Cx36 enrichment in selectively vulnerable neuronal populations with the developmental peak of electrical coupling provides a mechanistic framework for understanding why the immature TRN sustains disproportionate injury after perinatal asphyxia, and identifies connexin blockade as a rational, circuit-specific complement to existing therapeutic approaches.

## 6 Summary and future directions

The TRN occupies a critical position within thalamocortical circuits, serving as both gatekeeper regulating information flow to cortex and orchestrator of developmental oscillations essential for circuit maturation. Selective regional vulnerability is exposed within TRN intermediate and posterior segments which show pronounced injury after perinatal asphyxia or cardiac arrest in clinical settings and animal modeling, while anterior segments remain relatively preserved. This selective injury pattern correlates with the spatial distribution of developmental features concentrated in sensory TRN sectors: depolarizing GABAergic signaling maintained by low KCC2 expression, extensive Cx36-mediated electrical coupling, and strong excitatory drive from early-maturing FO thalamic nuclei. The temporal convergence of these developmental specializations, each independently implicated in hypoxic–ischemic injury pathways, accounts for the selective vulnerability of sensory TRN sectors during metabolic stress. The resulting cellular degeneration disrupts thalamocortical oscillations and impairs inhibitory gating, producing the persistent sensorimotor deficits and attentional impairments observed in survivors of neonatal hypoxic–ischemic encephalopathy.

Optimal therapeutic strategies likely require temporally precise interventions such as targeting the pathological phase of chloride dysregulation without impairing developmental GABA functions (Kadam and Hegarty, 2024; Pozzi et al., 2020). Combination therapy that synchronously reduces chloride import via NKCC1 inhibition and promotes chloride extrusion through KCC2 activation represents a rational next step for neuroprotection. The current generation of agents such as bumetanide, CLP290, and ANA12 collectively support the concept that pharmacologic stabilization of chloride homeostasis can mitigate excitotoxicity, restore inhibitory control, and potentially prevent secondary network remodeling following neonatal hypoxic–ischemic injury.

In parallel, therapeutic approaches targeting connexin and pannexin electrical coupling as adjuncts or alternatives to therapeutic hypothermia offer opportunities for restricting the cascade of circuit degeneration. The cell-type-specific and region-specific nature of connexin expression offers distinct advantages over hypothermia’s broad metabolic suppression. Many promising interventions identified in animal models face substantial hurdles for clinical translation, necessitating continued development of delivery technologies and treatment protocols optimized for human neonates. Nonetheless, the substantial progress in understanding TRN development, function, and vulnerability provides grounds for optimism that mechanism-based interventions targeting this critical structure may eventually improve outcomes for infants suffering hypoxic–ischemic brain injury.

### 6.1 Translational challenges and limitations of rodent models

Critical gaps remain in translating cellular mechanisms identified in laboratory studies into clinically useful therapies. Rodent models, while indispensable, present inherent limitations that must be considered when interpreting experimental results. The Rice–Vannucci and asphyxial cardiac arrest models have yielded important mechanistic insight, but rodents have lissencephalic cortices, accelerated maturation, and simplified injury patterns that differ fundamentally from the gyrencephalic human brain (Clancy et al., 2001; Semple et al., 2013). These differences extend beyond anatomy to include species-specific expression of connexins, distinct trajectories of chloride transporter maturation, and divergent thalamocortical network organization (Pressler and Auvin, 2013; Workman et al., 2013).

Large-animal models such as fetal sheep and neonatal piglets help bridge these gaps. These species reproduce key features of human neurodevelopment, including gyrencephalic architecture, white matter composition, and extended maturation timelines, providing a more accurate framework for testing neuroprotective strategies under clinically relevant conditions (Davidson et al., 2012a; Gonzalez and Ferriero, 2009; Gunn et al., 1997; Thoresen et al., 1997).

Complementary approaches using human induced pluripotent stem cell (iPSC)-derived thalamic organoids now offer direct access to human developmental mechanisms. These three-dimensional systems model early formation of TRN-like structures and permit investigation of human-specific features such as connexin-36 persistence, KCC2 maturation kinetics, and pharmacologic modulation of chloride homeostasis (Bhaduri et al., 2020; Cadwell et al., 2019; Miura et al., 2022; Nascimento et al., 2019). Integrating results from across multiple animal models and iPSC-based studies are necessary steps to overcome translational challenges and limitations.

### 6.2 Human neurophysiological and imaging correlates of network dysfunction

Second, understanding how graded injury severity produces different degrees of circuit disruption and functional impairment represents a fundamental mechanistic challenge. Modern multimodal neuromonitoring now enables validation of mechanisms derived from experimental models in human neonates, and offers real-time biomarkers that can guide timing and selection of therapeutic interventions. For example, combining conventional MRI findings with continuous EEG monitoring enhances prognostic accuracy, but predictive precision peaks at 48–72 h. Therapeutic hypothermia suppresses cortical activity and can mask the true extent of neuronal injury. However, EEG signals can reflect intrinsic brain function more accurately once cooling-related artifacts have stabilized, typically by 48 to 72 h after (Abend et al., 2011; Chock et al., 2023).

Additional integration of near-infrared spectroscopy with amplitude-integrated EEG further improves identification of infants likely to recover without major brain injury. This combined approach provides a dependable window for evaluating network integrity and predicting long-term neurodevelopmental outcome; when both modalities appear normal at 54 to 60 h after birth, more than 95% of infants avoid significant brain injury on subsequent MRI imaging (Chock et al., 2023). In contrast, EEG abnormalities reliably identify infants at high risk for poor outcomes. Persistent burst-suppression patterns beyond 48 h, absence of sleep–wake cycling by 120 h, and high electrographic seizure burden (affecting 31–64% of cooled neonates) are signs of disrupted thalamocortical oscillations and impaired reticular–relay interactions (Chock et al., 2023; Glass et al., 2024). These EEG patterns are consistent with impaired maturation of oscillatory activity normally governed by reciprocal thalamic–reticular interactions (see Section 2.2). However, burst-suppression and absent sleep–wake cycling represent system-level failures that could theoretically arise from cortical pathology, relay neuron dysfunction, or reticular injury. The selective preservation of cortical architecture in many affected neonates, combined with documented thalamic injury on imaging, supports a subcortical locus of dysfunction, but cannot definitively isolate TRN-specific contributions without direct reticular recordings in human patients. Table 2 summarizing above mentioned evidence types and their interpretive constraints for conceptual precision.

**Table 2:** Evidence types and their interpretive constraints.

Source	Data type	Results	TRN specificity	Interpretive constraint
Ross and Graham (1993)	Human autopsy	Selective TRN neuronal loss (3/6 pediatric, 11/22 adult cases)	Direct	Small sample size; cannot determine if TRN loss is primary or secondary; survival times variable
Ton et al. (2021)	Rat CA histology	Segment-specific TRN degeneration (intermediate/posterior sectors)	Direct	Single time point (24 h); severe injury paradigm; does not address sublethal injury
Shoykhet et al. (2012) and Middleton et al. (2017)	Rat CA physiology	VPM hyperexcitability, TRN microglial activation	Indirect	Relay neuron measurements; TRN involvement inferred from network dysfunction and inflammation
Multiple studies	Human MRI	Thalamic volumetric loss, VL/posterior nuclei injury	Indirect	Cannot resolve TRN; general thalamic pathology
Multiple studies	Clinical EEG	Burst-suppression, absent cycling, loss of spindles	Indirect	System-level dysfunction; TRN contribution inferred from known oscillatory mechanisms
Multiple experimental	Connexin/KCC2	Neuroprotection with Cx36 blockade or KCC2 enhancement	Mechanistically relevant	Not TRN-specific; effects multiple brain regions

Hierarchy of evidence mentioned in review for TRN involvement in hypoxic–ischemic encephalopathy are summarized. Direct evidence demonstrates anatomical TRN pathology; indirect evidence shows thalamocortical network dysfunction consistent with impaired TRN inhibitory control; mechanistically relevant evidence identifies vulnerability pathways present in developing TRN. The integration of these complementary approaches supports TRN involvement while acknowledging that definitive causal links require future TRN-specific experimental interrogation.

The developmental biology of the TRN provides a parsimonious explanation for its selective vulnerability and the resulting network pathology. Immature TRN neurons combine three mechanistically convergent features: depolarizing GABA responses that sustain rather than suppress excitation during metabolic stress (Bazhenov et al., 1999), inadequate KCC2-mediated chloride extrusion (Ulrich and Huguenard, 1997), and extensive connexin-36 electrical coupling that synchronizes and propagates calcium-mediated injury (Landisman and Connors, 2005). Experimental models highlighted in this review predict that when TRN inhibitory gating fails under these conditions, thalamocortical networks exhibit the burst-suppression, absent sleep–wake cycling, and attenuated spindle activity that characterize severe hypoxic–ischemic encephalopathy and predict poor neurodevelopmental outcomes.

The causal mapping between these subcellular mechanisms and specific clinical EEG signatures remains incompletely resolved, as current evidence derives primarily from system-level observations (clinical neuromonitoring, thalamic relay recordings) rather than direct TRN neuronal measurements in injured brains. Translating cellular insights into clinical interventions requires TRN-selective experimental manipulations and development of noninvasive biomarkers capable of isolating reticular contributions to network dysfunction in living patients.
